# Metabolomics Integrated with HPLC–MS Reveals the Crucial Antioxidant Compounds of Muscadine Wine

**DOI:** 10.3390/antiox12010055

**Published:** 2022-12-27

**Authors:** Fei Xue, Bohan Yang, Peining Fu, Yachun Peng, Jiang Lu

**Affiliations:** Center for Viticulture and Enology, School of Agriculture and Biology, Shanghai Jiao Tong University, Shanghai 200240, China

**Keywords:** muscadine grape, wine, antioxidant, pyrogallol

## Abstract

Wine is a kind of beverage with a variety of compounds beneficial to human health, which makes it popular all over the world and it contributes importantly to economics. The excessive oxidation of wine has always been a major problem in wine production and storage. Unlike traditional wines which are made from Eurasian grapes, wines made from muscadine grapes (*Muscadinia rotundifolia* Michx.) can maintain their sensory qualities under natural oxidation conditions for relatively long periods of time despite the insight mechanisms still being unclear. In this study, two muscadine wines, Carlos (CAL) and Noble (NOB), and two traditional wines, Chardonnay (CH) and Marselan (MAS), were chosen for comparison of their compositional alteration during oxidation, in order to analyze the principal components contributing to the antioxidant characteristics of muscadine wines. The DPPH, ORAC, color intensity, and total phenolic content changes during the natural oxidation process were analyzed. Six core significantly changed metabolites (SCMs, avicularin, beta-lactose, delphinidin-3-O-glucoside, ellagic acid, myricetin, and 4-methylcatechol [*p* < 0.05]) related to the oxidation process were determined. In addition, HPLC–MS was also used to identify pyrogallol which is a unique antioxidant compound in muscadine wine. The present work aims to reveal the crucial antioxidant compounds of muscadine wine and provide valuable information and a new platform for future research on wine oxidation.

## 1. Introduction

Wine is one of the most important alcoholic beverages in the world with thousands of years of history. High-quality wine not only has a rich aroma and a mellow taste, but it also has a variety of nutrients that are beneficial to human health, such as amino acids, vitamins, and phenolic substances [1,2,3,4,5]. With people’s pursuit of health and quality of life, wine consumption is growing rapidly all over the world. In 2019, 25.77 million tons of wine were produced worldwide, of which 23.75 million tons were consumed, leaving about 2 million tons in storage [6]. This portion of the stored wine will gradually deteriorate during oxidation.

Humans have known about wine oxidation for more than 100 years. In recent decades, researchers have completed more in-depth studies on the role and mechanism of wine oxidation [7,8,9]. It is generally believed that a moderate amount of oxygen plays positive effect on wine ageing, makes it more drinkable, and increases its drinking value [10]. This process brought by oxygen is called oxygenation [11]. However, excessive oxygen can negatively affect wine quality with various traits such as color browning and luster lost, fresh fruit flavor weakening or disappearance, and even the emission of an unpleasant aroma such as rotten apples, boiled cabbage, an acetic acid taste, etc., as well as being drier and bitter in terms of mouth-feel [12,13,14,15]. This negative process is called oxidation or being oxidized. Oxidation will affect the drinking value of the wine. Thus, control of oxidation is crucial to the quality of wine during production and storage. The way to control the rate of oxidation has always been an important issue of concern for wine producers and managers all over the world.

The grape varieties (*Vitis vinifera* L.) used in the modern wine industry are relatively easy to be oxidize. Therefore, large amounts of sulfur compounds such as SO_2_ are routinely added in production. However, this practice has been known as potentially harmful to wine quality and human health and therefore alternative treatments are needed. Muscadine grape (*Muscadinia rotundifolia* Michx.) is native to the southeast of the United States. Compared with *V. vinifera* grapevines, muscadine vines are very different in terms of growth habits and fruit characteristics [16,17]. Previous studies have shown that muscadine berries are rich in phenolic compounds; furthermore, it has also been found that muscadine berries contain ellagic acid and ellagitannin, which are absent in V. vinifera varieties [18]. It is generally believed that higher levels of polyphenols can lead to greater antioxidant activity [19]. Interestingly, despite the fact that muscadine wine is found to maintain a stable flavor and quality for a longer time after opening the bottle [20], its phenolic concentration has not been found to be significantly higher than traditional wine which is made from the *V. vinifera* grape. In recent years, muscadine fruit and wine have attracted a great deal of attention by growers and consumers because of its characteristics of stress tolerance and disease resistance, unique flavor, and rich aroma [21]. It could be a feasible solution to prevent oxidation by using the excellent natural antioxidant activity of muscadine grapes as either a raw material or the source of a functional extract in this connection. It will be valuable to profile the composition and utilize it through muscadine wine oxidation by using cutting-edge techniques. In this study, berries of two *M. rotundifolia* grape and two *V. vinifera* grape varieties were chosen for wine production with the same standard fermentation process; the physicochemical indices were analyzed as the quality control of fermentation. The antioxidant differences were compared by using various antioxidant-related attributes, and metabolomics analysis was conducted to comprehensively profile the compositional changes during the oxidation process of wine. In the conclusion, multivariate data analysis was used to determine the principal components contributing to potentially prevent the oxidation of muscadine wine.

## 2. Materials and Methods

### 2.1. Field Conditions and Materials

The grape materials used in this research were from four grape varieties: *Vitis vinifera* ‘Chardonnay’ (CH), *Vitis vinifera* ‘Marselan’ (MAS), *Muscadinia rotundifolia* ‘Carlos’ (CAL), and *Muscadinia rotundifolia* ‘Noble’ (NOB).

‘CH’ and ‘MAS’ were spaced at 2.75 m × 1.5 m with stock, drip-irrigated with no rain shelter, managed through traditional management, and were planted in the vineyard at Domaine Franco-Chinois (Huailai county, Zhangjiakou city, Hebei province, China) in 2014. The vineyard is located at 40.35° N, 115.83° E, the altitude is 520 m, the soil type is gravel sandy loam, the average annual rainfall is 413 mm, and the frost-free period is 149 d.

‘CAL’ and ‘NOB’ were spaced at 2.5 m × 1 m with no scion and stock, drip-irrigated, covered by a simple rain shelter, managed through traditional management, and were planted in the germplasm resources vineyard at the Center for Viticulture and Enology of Shanghai Jiao Tong University (Minhang district, Shanghai city, China) in 2016. The vineyard is located at 31.04° N, 121.45° E, the altitude is 22 m, the soil type is mucky clay, the average annual rainfall is 1166.1 mm, and the frost-free period is 230 d.

When all of the grape materials were fully ripe in September 2019, the grapes were picked at random from the whole bunch, and then immediately entered into into the vinification process.

### 2.2. Vinification

‘CH’ and ‘CAL’ were used to make white wine in the following processes. Briefly, the grape berries were crushed by a roller crusher. The grape juice was extracted by a basket presser (pressure < 1.2 bar) and 40 mg/L of SO_2_ was added. After standing at 5 °C for 24 h, the clear juice (NTU < 100) was transferred into a 100 L steel fermenter. Commercial yeast ‘LALLEMAND-CY3079′ was added (200 mg/L), and the fermentation temperature was controlled at 16 ± 1 °C. After the end of alcohol fermentation, 40 mg/L SO_2_ was added and bottled until full.

‘MAS’ and ‘NOB’ were used to make red wine in the following processes. The grape berries were crushed by a roller crusher, and the crushed must was transferred into a 100 L steel fermenter and 40 mg/L of SO_2_ was added. Commercial yeast ‘LALLEMAND-BD254′ was added (200 mg/L) to the must, and the fermentation temperature was controlled at 26 ± 2 °C. After the end of alcohol fermentation, 40 mg/L of SO_2_ was added and the clear wine was extracted by a basket presser (pressure < 1.2 bar). After standing 24 h at 5 °C, the clear wine (NTU < 100) was bottled until full.

The bottled wine was stored at 8 °C in a dark environment for further analysis.

### 2.3. Determination of Physicochemical Indices of Wine

The soluble sugar (SS), titratable acid (TA), alcohol, total SO_2_, and free SO_2_ of the wine were determined by the method of OIV [22]. The pH was measured with a Mettler Toledo FE20 Desktop pH Meter (Mettler Toledo Instruments Co. Ltd., Shanghai, China).

### 2.4. Oxidation Processes of Wine Samples

The wine samples were taken out of the bottle and divided into 50 mL centrifuge tubes. These tubes were covered by plastic film with air holes and naturally oxidized in a constant temperature incubator (25 °C) to avoid light for six days. Every wine sample included at least seven tube replicates. On each day of the oxidation processes, one tube from each wine sample was taken out for further analysis.

### 2.5. Wine Colour Measurement

The indexes of wine color were determined by a published method with modification [23]. Briefly, a spectrophotometer (V1100D, MAPADA, Shanghai, China) was used to determine the absorbance of the wine samples at 420, 520, and 620 nm using a 0.2 cm path-length quartz cuvette (A_420_, A_520_ and A_620_, respectively). The color density (CD) was the sum of A_420_ and A_520_; the color intensity (CI) was the sum of A_420_, A_520_, and A_620_; tint T was the ratio of A_420_ to A_520_.

### 2.6. Determination of Antioxidant Indices

The DPPH (2,2-diphenyl-1-picrylhydrazyl) and ORAC (oxygen radical absorbance capacity) assays were conducted to test the antioxidant capacity of the wine samples.

The DPPH scavenging capacity of the wine samples was analyzed by a modified method according to the Chrzczanowicz et al. [24]. The four samples were diluted with water to different folds to meet the experimental requirements (CH: 2-fold, CAL: 10-fold, MAS: 25-fold, and NOB: 25-fold). A total of 5 μL of diluted the wine sample and 195 μL of methanolic DPPH solution (25 mg/L) were added to a 96-well microtiter plate. The system reacted in darkness for 40 min at 25 °C. A 10% ethanol solution was used as a blank control. The absorbance at 515 nm was detected using a multi-mode microplate reader (Synergy2, Biotek, Winooski, VT, USA). The calculation formula was scavenging activity (%) = (1-A_515_sample/A_515_blank) × 100%. The DPPH scavenging capacity was expressed as Trolox equivalents (mMol/L).

The ORAC assay was conducted by a modified method according to Huang et al. [25]. The wine samples were diluted 500 times in phosphate buffer (75 mMol/L, pH = 7.4). A total of 25 μL of the diluted wine sample, 150 μL of fluorescein (4 μMol/L), and 25 μL of AAPH solution (160 mMol/L) were added to a 96-well microtiter plate. The reaction condition was set as follows: the excitation wavelength was 485 nm, the emission wavelength was 535 nm, the reaction temperature was 37 °C, and the cycle period was 120 min. The multi-mode microplate reader (Synergy2, Biotek, Winooski, VT, USA) was used to monitor the reaction until 95% fluorescence loss was achieved. The 25 μL of PBS buffer was used instead of the sample as a blank control. The area under the curve (AUC) was calculated by the approximate integral method: AUC = 0.5 + (R_2_/R_1_) + (R_3_/R_1_) + (R_4_/R_1_) + … + 0.5 (R_n_/R_1_). The ORAC was expressed as Trolox equivalents (mMol/L).

### 2.7. Total Phenolic Content, HPLC–MS, and Metabolomic Analysis

The total phenolic content (TPC) was measured by the Folin–Ciocalteu colorimetric method. The four samples of wine were diluted, and then Folin–Ciocalteu reagent and sodium carbonate were successfully added. The mixed solution was reacted at 40 °C for 30 min. A spectrophotometer (V1100D, MAPADA, Shanghai, China) was used to determine the absorbance of the solution at 760 nm. The TPC was expressed as the gallic acid equivalent (mg/L).

The HPLC–MS analysis of phenolic compounds was performed using a high-performance liquid chromatography-mass spectrometry (HPLC–MS) system (VION IMS QTof, WATERS, Milford, MA, USA). HPLC separation was performed on a reversed phase C18 column (2.1 mm × 100 mm × 1.7 μm). The conditions of HPLC were as follows: the column temperature was 45 °C, phase A was 0.1% formic acid solution, phase B was 0.1% formic acid—acetonitrile solution, and the flow rate was 0.4 mL/min. The electrospray ion source was set to negative ion mode. The conditions of MS were as follows: the source temperature was 450 °C, the ionspray voltage was 2 kV, the collision energy was 40 ± 10 eV, the ionspray flow rate was 15 L/min, and the range of scanning was 50–1000 *m*/*z*. The phenolic compounds were identified based on the total ion chromatogram, exact molecular weight, retention time, and fragmentation characteristics.

The metabolomics analysis was performed by Biotree Co., Ltd. (Shanghai, China) using the samples at 0, 3, and 6 days of the oxidation process.

### 2.8. Statistical Analysis

All data in this research were analyzed using SPSS Statistics software (V24.0, IBM SPSS, Chicago, USA), and the results were expressed as the mean ± standard deviation (SD) of triplicates. The differences between the means were analyzed using Duncan’s multiple range test and the *p* < 0.05 was considered to be significant. Correlation heat maps were generated using the MetaboAnalyst website (http://www.metaboanalyst.ca/, accessed on 22 June 2022).

## 3. Results

### 3.1. Characteristics of Muscadine Wines

In this study, physicochemical indices were measured as the quality-control for the winemaking process. Four wine samples used in this research were tested for soluble sugar (SS), titratable acid (TA), alcohol, total SO_2_, free SO_2_ contents, and pH, as shown in Table 1.

The SS contents of these four wine samples are listed from low to high as 1.12 g/L for CAL, 1.35 g/L for CH, 2.34 g/L for NOB, and 2.81 g/L for MAS, respectively, in which the first two are white and the last two are red. Since the SS content of all four wines are less is 4 g/L, they are classified as dry wines. Sugar content has long been an indicator of wine grape ripeness. During fermentation, most of the sugar was converted into alcohol and CO_2_ by yeast. However, there was still SS content left in the wine after fermentation. The TA content in CH wine, which reached to 6.32 g/L, was the highest among the samples in this study. In muscadine wine, the TA contents were 5.39 g/L and 5.11 g/L, respectively, and were lower than the same color type of traditional wine.

The alcohol content of traditional wines could reach 12–15%; the alcohol contents of the CH and MAS wines fit into this range. However, the muscadine wine was well below this range due to its low soluble sugar content in fruits. The alcohol contents of CAL wine and NOB wine were 7.52% and 7.04%, respectively, and were almost half that of the traditional wine.

The total SO_2_ contents of the samples were between 70.12 to 92.19 mg/L, and the free SO_2_ contents were between 23.33 to 32.21 mg/L. The difference in total and free SO_2_ contents were obviously consistent between the samples. The appropriate content of SO_2_ in the wine has positive effects on wine quality, such as preventing oxidation, improving microbial stability, maintaining stable acidity, and accelerating clarification. Generally, SO_2_ or sulfur compounds are often added manually during the wine-making process or bottling. However, excessive SO_2_ in wine brings about an unpleasant smell and even poses risks to human health. The International Organization of Vine and Wine (OIV) has therefore set up a standard that requires the total SO_2_ in wine to be approximately 200 mg/L and the free SO_2_ ≤ 50 mg/L was considered in the wine. Hence, the SO_2_ of the four samples in this research were within the reasonable range.

The pH was usually negatively correlated with TA; the test results were also consistent [26]. Among the four samples, the pH of NOB was the highest at 3.68, while the pH of CH was 3.32, which was the lowest among the four.

All these indices were in the normal range of wine, which showed that there were no quality problems in the four samples. The differences between these indices indicated that different grape materials, especially the wines made from muscadine grapes, had particular characteristics in comparison with the traditional wines made from *V. vinifera* grapes.

### 3.2. Changes in Relevant Attributes during the Oxidation Process

#### 3.2.1. DPPH and ORAC

In previous studies by our research group, we noticed that muscadine wines could retain their original sensory characteristics when they were exposed to air for 2 weeks. The oxidation features found in traditional wines, such as browning, a rotten smell, and acetification, were not found in muscadine wines. These phenomena suggested that muscadine wines possess excellent antioxidant properties. An experiment was therefore conducted to investigate the antioxidant attributes under oxidation treatment. The DPPH and ORAC were chosen to represent the changes in the antioxidant indices during wine oxidation.

**Figure 1:**
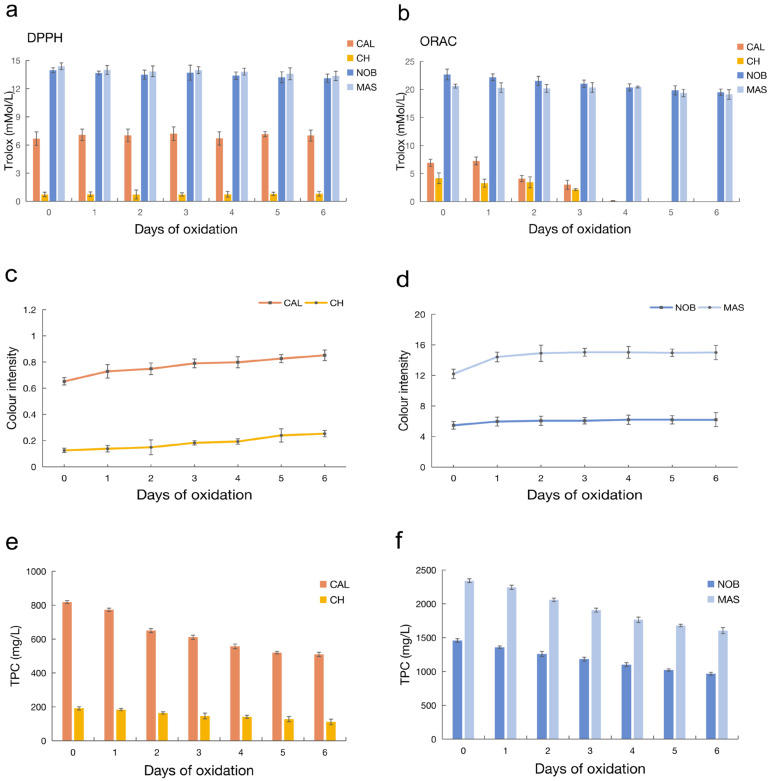
Antioxidant indices, color intensity, and total phenol contents of the wine during oxidation. (**a**) The DPPH of four wine samples during oxidation; (**b**) the ORAC of four wine samples during oxidation; (**c**) color intensity of the CAL and CH wines; (**d**) colour intensity of the NOB and MAS wines; (**e**) the TPC of CAL and CH; and (**f**) the TPC of NOB and MAS.

All of the wine samples in centrifuge tubes were kept in the dark in an incubator at 25 °C for six days. Before oxidation (0 d), the DPPH was 6.67 for CAL, 0.73 for CH, 13.96 for NOB, and 14.38 for MAS (mMol/L, Trolox), respectively (Figure 1a). The DPPH of CAL was consistently about nine times higher than that of CH throughout the oxidation process. MAS was slightly higher than NOB at each time point, but the difference was not significant. As oxidation proceeded, little change was found in the CAL and CH wines while there was a slight decrease in NOB and MAS wines. As for the ORAC, before oxidation (0 d), the ORAC was 6.92 for CAL, 4.18 for CH, 22.66 for NOB, and 20.6 for MAS (mMol/L, Trolox), and the differences between CAL vs. CH and NOB vs. MAS were significant (Figure 1b). The ORAC of all of the wines decreased gradually as oxidation proceeded. However, after oxidation exposure for 2 days, the differences between the two samples in each pair became no longer significant. On day 4, the ORAC values of CAL and CH were decreased to about 0 mMol/L (Trolox). Overall, the antioxidant indices of red wine were significantly higher than those of white wine, and the indices gradually decreased as the oxidation time was prolonged.

#### 3.2.2. Color Intensity

Color change is an intuitive phenomenon in the oxidation process of wine. In this research, color intensity (CI) was used to show the changes in the color of the wine (Figure 1c,d). However, the CI was affected by many factors, such as anthocyanin content, acidity, and other variety factors. Therefore, the difference in CI between 6 d and 0 d (CI change rate) of each sample were further analyzed (Figure S1). The CI change rates of each sample were 30.52%, 102.40%, 13.47%, and 22.95% for CAL, CH, NOB, and MAS, respectively. The CI change rate of traditional wine was significantly higher than that of muscadine wine with the same color. The muscadine wines were obviously more stable during the oxidation process. This result was also consistent with our previous experience.

#### 3.2.3. Total Phenolic Content

The total phenolic content (TPC) of each sample during oxidation was measured before and during the oxidation process. The TPC of CAL was about 4.27 times higher than CH before the oxidation process and this difference between the two remained about the same at different time points during oxidation although they both decreased (Figure 1e,f). This result was consistent with the antioxidant indices. It is generally believed that TPC is positively correlated with antioxidant capacity. However, the TPC in NOB and MAS was contradictory to the antioxidant indices, although the TPC of MAS was higher than NOB during the entire oxidation process.

It is generally believed that the higher TPC, the higher the antioxidant indices values and the lower the CI change rate during the oxidation process. However, in this research, NOB with lower TPC had higher ORAC values and lower CI change rates. Compared with CH, the DPPH multiple of CAL was also much higher than the TPC multiple. Based on the contradictions presented in these results, we speculated that there might be more potent antioxidants in muscadine wine. Thus, metabolomic analysis has been implemented for these speculations in later work.

### 3.3. Metabolomic Analysis of Wine Samples during Oxidation

**Figure 2:**
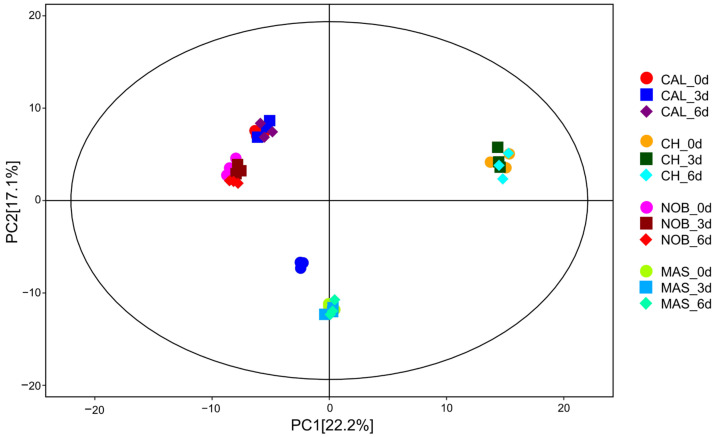
PCA score plots of the metabolite profiling data of the wine samples.

The metabolite profiles of the wine samples were obtained by UHPLC–Q-Orbitrap-HRMS-based metabolomics. The features of 1644 metabolites were detected, and normalized data were saved as a CSV format file (Figure 2). SIMCA software was used to conduct principal component analysis (PCA). The overview of the groupings and outliers can be obtained from the PCA, and two PCs explained more than 39.3% of the total variance (PC1 and PC2 explained 22.2% and 17.1%, respectively). The plots of all the samples were within the 95% Hotelling’s T-squared ellipse. The plots of each group were clearly separated and the QCs were clustered together, which showed the reproducibility and stability of the metabolomics method [27]. OPLS-DA was used to compare the metabolomics data between ‘CAL vs. CH’ and ‘NOB vs. MAS’ on the same day (Figure S2). Therefore, the OPLS-DA models were suitable to confirm the differences between the groups [28].

**Figure 3:**
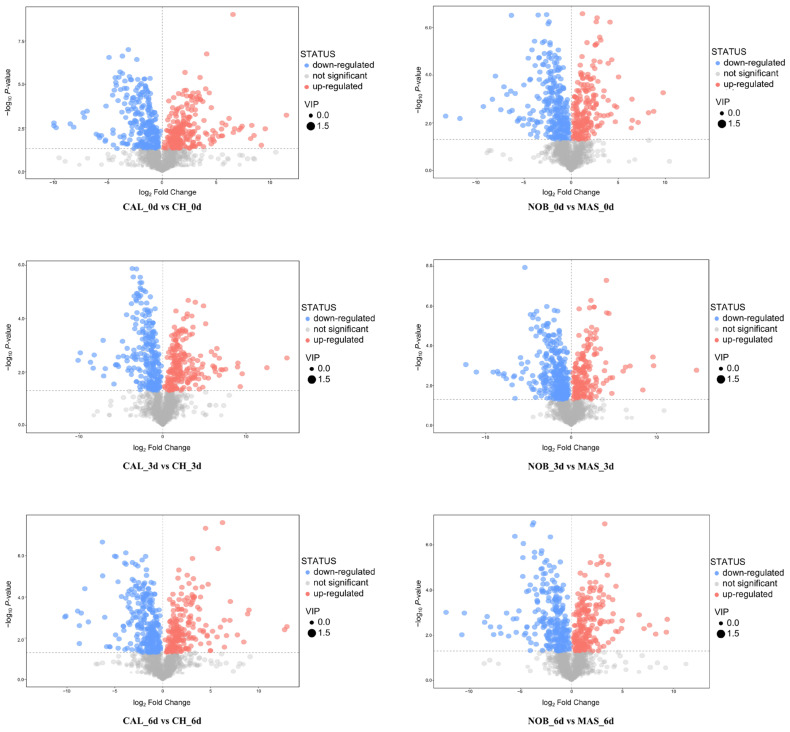
SCMs in different comparison groups during the oxidation process.

A combination of two conditions was used to select the significantly changed metabolites (SCMs) between the groups: the *p*-value of the Student’s *t*-test < 0.05, and the variable importance in the projection (VIP) of PC1 in the OPLS-DA model > 1. Each plot in the volcano plots represented a metabolite, the abscissa represented the logarithm of the multiple change of the metabolites, the onate represents the *p*-value of the *t*-test, and the plot size represented the VIP values of the OPLS-DA model which were bigger with a larger size (Figure 3). The upregulated SCMs were shown in red, the downregulated SCMs were shown in blue, and the non-significantly different metabolites were shown in gray. Metabolites only present in CAL or NOB were classified as upregulated and those only present in CH or MAS were classified as downregulated. The number of upregulated SCMs was 198-249, and the number of downregulated SCMs was 254–320 across all of the comparison groups during the oxidation process. A large number of SCMs were identified at all stages of the oxidation process, and the number of downregulated SCMs was slightly more than that of the upregulated SCMs, which was basically consistent in all of the comparison groups. These SCMs showed a normal distribution in the number of different fold changes (FC). While the vast majority of SCMs differed in content by less than 10 times, a few SCMs differed by more than 100 times (Figure S3). It was worth noting that some metabolites were much more abundant in muscadine wine than in traditional wine in each comparison group, such as myricitrin, pyrocatechol, lupeol acetate, and quercitrin. Those metabolites present only in muscadine wine before oxidation received additional attention. For example, oxysanguinarine, troxerutin, crocin, k-strophanthoside, chebulagic acid, luteolinidin chloride, beta-carotene, and goshonoside F1 were detected only in CAL but not in CH, and ricinoleic acid and ganoderic acid D2 were detected only in NOB but not in MAS.

These SCMs were enriched in the KEGG metabolic pathway, and the rich factor was calculated based on the ratio of annotated SCMs to total metabolites in the pathway. The abscissa represented the rich factor corresponding to each pathway; the larger the plot means the more metabolites and the redder the color means the more significant the enrichment degree. The SCMs in ‘CAL vs. CH’ were mainly enriched in metabolic pathways, amino acids biosynthesis ABC transporters, cofactors biosynthesis, and flavonoid biosynthesis. The enriched results of SCMs in ‘NOB vs. MAS’ were similar (Figure S4). Analysis of the KEGG metabolic pathway indicated that the differential metabolites between CAL and CH, as well as between the NOB and MAS wine samples, were similar in KEGG classification.

### 3.4. Selection of Common SCMs Associated with Antioxidant Capacity in Wine

**Figure 4:**
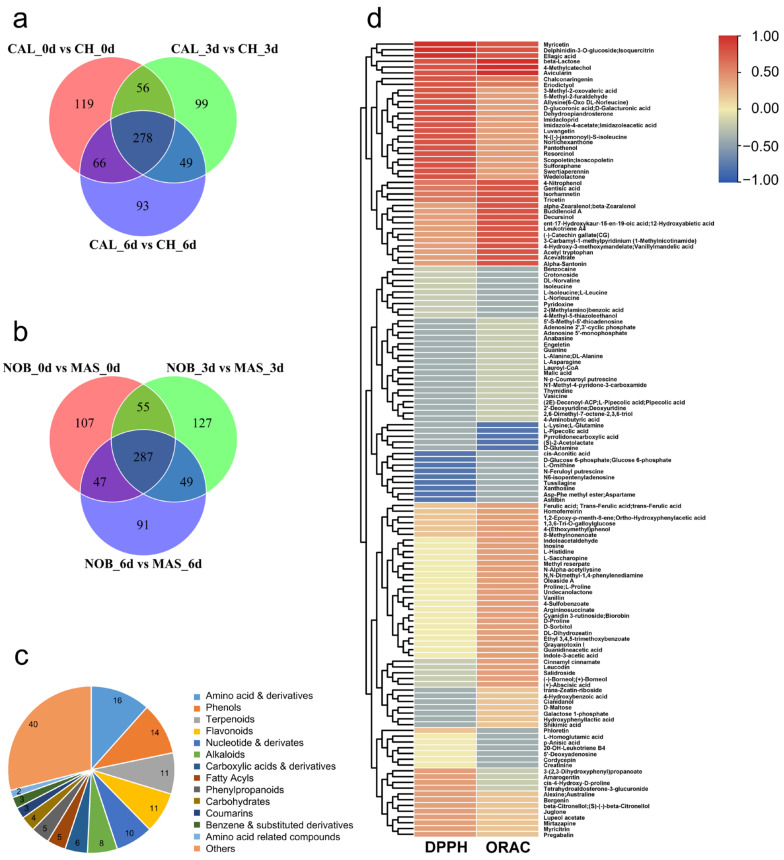
Selection of antioxidant-related compounds from SCMs. (**a**) Venn analysis of the SCMs in CAL vs. CH during oxidation; (**b**) Venn analysis of the SCMs in NOB vs. MAS during oxidation; (**c**) classification of the core 138 SCMs between CAL vs. CH and NOB vs. MAS; (**d**) and correlation heat map of core the 138 SCMs and antioxidant indices.

The Venn analysis revealed the difference and coincidence of SCMs in the different stages of the oxidation process (Figure 4a,b). According to the classification of SCMs in each comparison group, the proportion of various metabolite classes in the total SCMs between muscadine wine and traditional wine can be seen. Within each group, flavonoids, alkaloids, phenols, amino acids, and derivatives were the largest proportion (Figure S5). Among them, flavonoids and phenols have been well recognized as possessing antioxidant properties. There was a total of 278 SCMs posting differences between the CAL and CH samples at 0, 3, and 6 d. Similarly, a total of 287 SCMs appeared in the comparison between the NOB and MAS samples. Among the different metabolites identified in these two groups, 138 of them appeared at all stages during the oxidation process of ‘CAL vs. CH’ and ‘NOB vs. MAS’. The classification and quantity of these common 138 SCMs was analyzed, of which 16 metabolites were classified into amino acids and derivatives, 14 were phenols, 11 were terpenoids, and 11 were flavonoids (Figure 4c). It has been well documented that amino acids affect the wine volatile composition [29,30]. Terpenoids could form the axis for the sensory expression of wine [31]. The great difference in amino acids and terpenoids might be the reason for the great aroma difference between muscadine and traditional wines. Many SCMs were phenols and flavonoids, which are known to contribute to antioxidation.

Analysis between the 138 core SCMs and antioxidant indices was performed and a heat map was constructed (Figure 4d). Avicularin, beta-lactose, delphinidin-3-O-glucoside (isoquercitrin), ellagic acid, myricetin, and 4-methylcatechol had a very high positive correlation with both DPPH and ORAC. Chalconaringenin, eriodictryol, gentisic acid, isorhamnetin, tricetin, and 4-nitrophenol also showed some degree of positive correlation with the antioxidation indices. In the chemical structure, most of these SCMs had important antioxidant groups such as phenolic hydroxyl groups. They were likely the key contributors to the excellent antioxidant capacity of muscadine wines. As the core metabolites responsible for the differences in sensory and antioxidant capacity between muscadine and traditional wines, these 138 metabolites in muscadine wines deserve additional studies in further detail.

### 3.5. Pyrogallol Was a Unique Antioxidant Compound in the Muscadine Wine Samples

**Figure 5:**
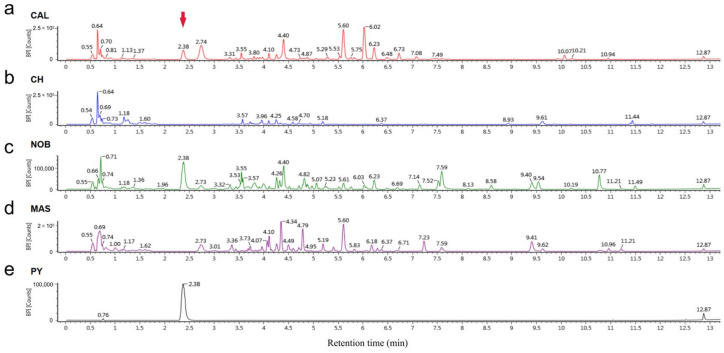
HPLC chromatograms of wine and pyrogallol solution. (**a**) CAL; (**b**) CH; (**c**) NOB; (**d**) MAS; (**e**) Pyrogallol. HPLC separation: reversed phase C18 column (2.1 mm × 100 mm × 1.7 μm); temperature: 45 °C; phase A: 0.1% formic acid solution; phase B: 0.1% formic acid—acetonitrile solution; flow rate: 0.4 mL/min. MS source temperature: 450 °C; ionspray voltage: 2 kV, flow rate: 15 L/min, scan range: 50–1000 *m*/*z*.

HPLC analysis was performed to detect the different compounds that existed in the muscadine wine but were absent in the traditional wine. By comparing the chromatograms, a significant peak present only in CAL and NOB, but not in CH and MAS, was noted. In this study, the retention time of this peak was at 2.38 min, and it was consistent across the samples (Figure 5a–d). According to the mass spectrum information of this peak, an online website was used for prediction and the specific information of this compound was finally confirmed. The compound was pyrogallol (1,2,3-Trihydroxybenzene, C_6_H_6_O_3_, KEGG Entry: C01108) and its chemical structure was a benzene ring linked with three adjacent phenolic hydroxyl groups (Figure S6a).

Pyrogallol is an odorless white to gray solid, soluble in water, ethanol, and ether. According to the HPLC chromatogram and the mass spectrum information of the corresponding peaks of pyrogallol solution detected under the same conditions, the information was consistent with the CAL and NOB samples (Figure 5 and Figure S6b–d). By quantification of the target peak, the contents of pyrogallol in CAL and NOB were 59.22 and 286.10 mg/L, respectively (Figure S7).

**Figure 6:**
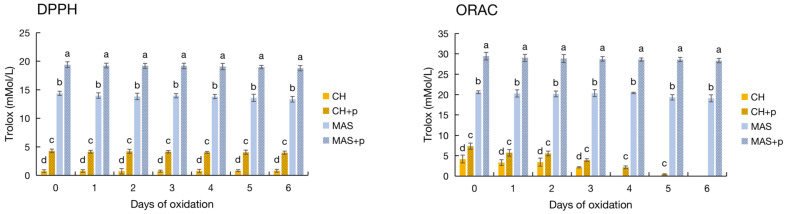
Antioxidant indices of Chardonnay and Marselan wines with pyrogallol during oxidation. Different letters indicate significant differences among values at the same date using Duncan’s test (*p* ≤ 0.05).

Since pyrogallol was only found in the CAL and NOB muscadine wines, a similar amount of pyrogallol was added to the CH and MAS wines to determine their antioxidant activities. The DPPH increased from 0.73 to 4.28 mMol/L (Trolox) in the CH wine, and from 14.38 to 19.39 mMol/L (Trolox) in the MAS wine. The antioxidant capacity (DPPH) increased by 5.86 and 1.35 times, respectively (Figure 6). Similarly, the ORAC of CH increased from 4.18 to 7.35 mMol/L (Trolox) and that of MAS increased from 20.60 to 29.43 mMol/L (Trolox), which was 1.76 and 1.43 times that of the control. During the oxidation process, the DPPH and ORAC of all of the samples gradually decreased, but the samples added with pyrogallol decreased slowly and gently. At day 6 of oxidation, the DPPH for CH was 0.81 for the control and 3.98 for pyrogallol plus. Similarly, the DPPH of the original MAS was 13.36, and its value increased to 18.79 in pyrogallol-added wine (mMol/L, Trolox). As for the ORAC, the original CH decreased to 0 on days 4 while the ORAC value remained at 2.13 at days 4 and 0.42 (mMol/L, Trolox) at days 5 d. At 6 d of oxidation, the ORAC was 0 for CH and CH with pyrogallol, 19.10 for MAS, and 28.33 for MAS with pyrogallol (mMol/L, Trolox). The increase in antioxidant indices suggested that pyrogallol, contributed highly to the antioxidant capacity in muscadine wines.

## 4. Discussion

Many studies have confirmed that the characteristics of muscadine wines are different from traditional wines. For example, sugar and acid content are closely related to wine quality [32]. It is generally believed that a higher acidity requires a higher sugar content to achieve a balanced taste. Sugar content has long been an indicator of wine grape ripeness. During fermentation, most of the sugar was converted into alcohol and CO_2_ by yeast. However, there was still SS content left in the wine after fermentation. In this research, the SS contents of red wines were significantly higher than that of white wines, and the SS contents of traditional wine (CH and MAS) were higher than that of muscadine wines (CAL and NOB). Sugar in grapes and wine is mainly composed of glucose and fructose, and some varieties have a small amount of sucrose [33,34]. An appropriate TA content gives a wine a refreshing flavor. The TA in traditional wine is composed of organic acids such as tartaric acid, malic acid, and citric acid, but in muscadine wine, the TA is composed of tartaric and succinic acid [32,35].

Oxidation is an inevitable process in wine production and storage. At room temperature and a standard atmospheric pressure, up to 8.6 mg/L O_2_ will be absorbed in oxidative wine [36]. O_2_ is a key factor for wine to achieve optimum quality [10]. According to the type of reaction, oxidation in wine can be divided into enzymatic oxidation and non-enzymatic oxidation. During the enzymatic oxidation process of wine, monophenol is oxidized to monocatechol and then eventually converted to quinone. Quinones polymerize with other phenols and proteins to produce brown precipitates, known as oxidative browning [12]. Non-enzymatic oxidation is also called chemical oxidation. Substrates such as catechin, epicatechin, and gallic acid are first oxidized to semiquinone and then oxidized to quinones [37]. The reduction of O_2_ to H_2_O_2_ is accompanied by the transformation of different forms of Fe^3+^/Fe^2+^ and Cu^2+^/Cu^+^ [38].

The phenolic compounds of wine are composed of flavonoids, flavanols, and anthocyanin. The differences in the content and composition of these compounds are the main reasons for the differences in wine taste [39]. The antioxidant capacity, and the reaction rate, depend on the chemical composition and structure of the phenolics and the stability of the oxidation products [7]. Some of the antioxidant mechanisms of phenols have been proposed. Carotenoids are effective in eliminating singlet oxygen [40]. Anthocyanins are mainly color compounds in wine; malvidin-3-glucoside in particular is a monomer with the largest proportion of anthocyanin in grapes [41] and can react with peroxynitrite to reduce oxidative damage [42]. In addition, flavonoid and baicalin also demonstrate strong antioxidant activity and have a direct reaction with peroxynitrite [43]. Previous studies have widely suggested that phenolics play a key role in the antioxidant process of wine [36].

As previously reported, pyrogallol-containing molecules are ubiquitous in the plant kingdom, but the chemical synthesis of these molecules is still challenging [44]. When grape seeds were roasted at 220 °C, pyrogallol could be obtained from hydrolysable tannins [45]. However, studies of this compound in grapes and wine are still lacking. From its chemical structure and previous research, pyrogallol has potent reducing properties [46]. Pyrogallol exhibited greater reactivity with Fe^3+^ in the absence of a nucleophile, suggesting that pyrogallol might be involved in the antioxidant process of wine by reacting with Fe ions and affecting the non-enzymatic reaction rate [47].

## 5. Conclusions

Wine oxidation is always a challenge in wine production and storage. Muscadine wines have better antioxidant capacity than traditional wines made from Eurasian grapes. In this research, two muscadine grapes and two Eurasian grapes (one red and one white of each) were used to make wine and their physicochemical indices were analyzed. During the natural oxidation process, the wine samples generally showed decreasing antioxidant indices (DPPH and ORAC), increasing color intensity, and decreasing total phenolic content, but the muscadine wines had smaller changes in the whole process with better stability in quality. Combined with metabolomics and HPLC detection, a large number of SCMs were identified, six of which were highly correlated to antioxidants; then, an antioxidant compound uniquely in muscadine wines named pyrogallol was identified.

This research has explained the high antioxidant capacity of muscadine wines and has provided additional information for the antioxidant mechanism of muscadine wines although many challenge remain to be answered. We suggest that pyrogallol deserves more attention in the research of wine and enology.

## Figures and Tables

**Table 1:** Physicochemical indices of wine.

Sample	Residue Sugar (g/L)	Total Acidity (g/L)	Alcohol (%)	Total SO_2_ (mg/L)	Free SO_2_ (mg/L)	pH
CAL	1.12 ± 0.09	5.39 ± 0.14	7.52 ± 0.24	78.24 ± 0.56	29.08 ± 0.87	3.63 ± 0.02
CH	1.35 ± 0.26	6.32 ± 0.08	13.53 ± 0.31	92.19 ± 1.34	32.21 ± 0.69	3.32 ± 0.02
NOB	2.34 ± 0.12	5.11 ± 0.17	7.04 ± 0.22	70.12 ± 1.83	23.33 ± 0.28	3.68 ± 0.03
MAS	2.81 ± 0.15	5.52 ± 0.11	13.98 ± 0.27	84.85 ± 1.02	30.04 ± 0.35	3.51 ± 0.01

## Data Availability

Not applicable.

## Supplementary Materials

The following supporting information can be downloaded at: https://www.mdpi.com/article/10.3390/antiox12010055/s1, Figure S1. Colour intensity change rate (CI-r) during oxidation; Figure S2. OPLS-DA score plots in different sample groups; Figure S3. Fold change distribution of SCMs in different comparison group; Figure S4. KEGG enrichment for different comparison groups; Figure S5. Classification of SCMs in different comparison groups; Figure S6. Mass spectrum information of wine and pyrogallol solution; Figure S7. Contents of pyrogallol in CAL and NOB wine sample.

## References

[1] Yang J., Martinson T.E., Liu R.H. (2009). Phytochemical profiles and antioxidant activities of wine grapes. Food Chem..

[2] Ferraz da Costa D.C., Rangel L.P., Quarti J., Santos R.A., Silva J.L., Fialho E. (2020). Bioactive compounds and metabolites from grapes and red wine in breast cancer chemoprevention and therapy. Molecules.

[3] Mazué F., Delmas D., Murillo G., Saleiro D., Limagne E., Latruffe N. (2014). Differential protective effects of red wine polyphenol extracts (RWES) on colon carcinogenesis. Food Funct..

[4] Nunes C., Ferreira E., Freitas V., Almeida L., Barbosa R.M., Laranjinha J. (2013). Intestinal anti-inflammatory activity of red wine extract: Unveiling the mechanisms in colonic epithelial cells. Food Funct..

[5] Ju Y.L., Yang B.H., He S., Tu T.Y., Min Z., Fang Y.L., Sun X.Y. (2019). Anthocyanin accumulation and biosynthesis are modulated by regulated deficit irrigation in cabernet sauvignon (*Vitis vinifera* L.) grapes and wines. Plant Physiol. Biochem..

[6] OIV (2019). 2019 Statistical Report on World Vitiviniculture. https://www.oiv.int/public/medias/6782/oiv-2019-statistical-report-on-world-vitiviniculture.pdf.

[7] Danilewicz J.C. (2003). Review of reaction mechanisms of oxygen and proposed intermediate reduction products in wine: Central role of iron and copper. Am. J. Enol. Viticult..

[8] Oliveira R., Marques J., Bento F., Geraldo D., Bettencourt P. (2011). Reducing antioxidant capacity evaluated by means of controlled potential electrolysis. Electroanalysis.

[9] Dimkou E., Ugliano M., Dieval J.B., Vidal S., Aagaard O., Rauhut D., Jung R. (2011). Impact of headspace oxygen and closure on sulfur dioxide, color, and hydrogen sulfide levels in a Riesling wine. Am. J. Enol. Viticult..

[10] Ugliano M. (2013). Oxygen contribution to wine aroma evolution during bottle aging. J. Agric. Food Chem..

[11] Sanchez-Gomez R., del Alamo-Sanza M., Martinez-Martinez V., Nevares I. (2020). Study of the role of oxygen in the evolution of red wine colour under different ageing conditions in barrels and bottles. Food Chem..

[12] Li H., Guo A., Wang H. (2008). Mechanisms of oxidative browning of wine. Food Chem..

[13] Pons A., Nikolantonaki M., Lavigne V., Shinoda K., Dubourdieu D., Darriet P. (2015). New insights into intrinsic and extrinsic factors triggering premature aging in white wines. Advances in Wine Research.

[14] Ferreira A.C.S., Oliveira C., Hogg T., de Pinho P.G. (2003). Relationship between potentiometric measurements, sensorial analysis, and some substances responsible for aroma degradation of white wines. J. Agric. Food Chem..

[15] Cullere L., Cacho J., Ferreira V. (2007). An assessment of the role played by some oxidation-related aldehydes in wine aroma. J. Agric. Food Chem..

[16] Xu X., Lu J. Cytogenetic study of interspecific hybrids between *Vitis rotundifolia* and *Vitis vinifera*. Proceedings of the 26th International Horticultural Congress.

[17] Talcott S.T., Lee J.H. (2002). Ellagic acid and flavonoid antioxidant content of muscadine wine and juice. J. Agric. Food Chem..

[18] Marshall D.A., Stringer S.J., Spiers J.D. (2014). Storage retention of stilbene, ellagic acid, flavonol, and phenolic content of muscadine grape (*Vitis rotundifolia* Michx.) cultivars. HortScience.

[19] Pastrana-Bonilla E., Akoh C.C., Sellappan S., Krewer G. (2003). Phenolic content and antioxidant capacity of muscadine grapes. J. Agric. Food Chem..

[20] Gris E.F., Mattivi F., Ferreira E.A., Vrhovsek U., Filho D.W., Pedrosa R.C., Bordignon-Luiz M.T. (2013). Phenolic profile and effect of regular consumption of brazilian red wines on in vivo antioxidant activity. J. Food Compos. Anal..

[21] Lamikanra O. (1987). Aroma constituents of muscadine wines. J. Food Qual..

[22] OIV (2021). Compendium of International Methods of Analysis-Oiv. https://www.oiv.int/public/medias/2492/oiv-ma-as312-03a-en.pdf.

[23] Yildirim H.K. (2006). Evaluation of colour parameters and antioxidant activities of fruit wines. Int. J. Food Sci. Nutr..

[24] Chrzczanowicz J., Gawron A., Zwolinska A., de Graft-Johnson J., Krajewski W., Krol M., Markowski J., Kostka T., Nowak D. (2008). Simple method for determining human serum 2,2-diphenyl-1-picryl-hydrazyl (DPPH) radical scavenging activity–possible application in clinical studies on dietary antioxidants. Clin. Chem. Lab. Med..

[25] Huang D.J., Ou B.X., Prior R.L. (2005). The chemistry behind antioxidant capacity assays. J. Agric. Food Chem..

[26] Esteban M.A., Villanueva M.J., Lissarrague J.R. (1999). Effect of irrigation on changes in berry composition of tempranillo during maturation. Sugars, organic acids, and mineral elements. Am. J. Enol. Viticult..

[27] Qu Q., Zhang Z., Li Y., Zhou Z., Ye Y., Lu T., Sun L., Qian H. (2019). Comparative molecular and metabolic responses of wheat seedlings (*Triticum aestivum* L.) to the imazethapyr enantiomers S-IM and R-IM. Sci. Total Environ..

[28] Chenguang Z., Yuanyuan T., Gossner S., Youfa L., Qingyao S., Engel K.H. (2019). Impact of crossing parent and environment on the metabolite profiles of progenies generated from a low phytic acid rice (*Oryza sativa* L.) mutant. J. Agric. Food Chem..

[29] Ertekin B., Okur O.D., Guzel-Seydim Z. (2009). Formation of flavor compounds by amino acid catabolism in cheese. Gida.

[30] Procopio S., Krause D., Hofmann T., Becker T. (2013). Significant amino acids in aroma compound profiling during yeast fermentation analyzed by PLS regression. LWT-Food Sci. Technol..

[31] Mateo J.J., Jimenez M. (2000). Monoterpenes in grape juice and wines. J. Chromatogr. A.

[32] Sun X.Y., Cheng X.H., Zhang J.Z., Ju Y.L., Que Z.L., Liao X., Lao F., Fang Y.L., Ma T.T. (2020). Letting wine polyphenols functional: Estimation of wine polyphenols bioaccessibility under different drinking amount and drinking patterns. Food Res. Int..

[33] Yang B.H., Yao H., Zhang J.X., Li Y.Q., Ju Y.L., Zhao X.F., Sun X.Y., Fang Y.L. (2020). Effect of regulated deficit irrigation on the content of soluble sugars, organic acids and endogenous hormones in Cabernet Sauvignon in the Ningxia region of China. Food Chem..

[34] Basha S.M., Vasanthaiah H.K., Kambiranda D.M., Easwaran K., Queeley G. (2012). Genetic variation in sugar composition among muscadine, florida hybrid bunch and bunch grape genotypes. Int. J. Wine Res..

[35] Lamikanra O. (1997). Changes in organic acid composition during fermentation and aging of noble muscadine wine. J. Agric. Food Chem..

[36] Singleton V.L. (1987). Oxygen with phenols and related reactions in musts, wines, and model systems: Observations and practical implications. Am. J. Enol. Viticult..

[37] Kilmartin P.A., Zou H.L., Waterhouse A.L. (2001). A cyclic voltammetry method suitable for characterizing antioxidant properties of wine and wine phenolics. J. Agric. Food Chem..

[38] Danilewicz J.C., Wallbridge P.J. (2010). Further studies on the mechanism of interaction of polyphenols, oxygen, and sulfite in wine. Am. J. Enol. Viticult..

[39] Cheynier V., Duenas-Paton M., Salas E., Maury C., Souquet J.-M., Sarni-Manchado P., Fulcrand H. (2006). Structure and properties of wine pigments and tannins. Am. J. Enol. Viticult..

[40] Boehm F., Edge R., Truscott T.G. (2012). Interactions of dietary carotenoids with singlet oxygen (^1^o_2_) and free radicals: Potential effects for human health. Acta Biochim. Pol..

[41] Yang B.H., He S., Liu Y., Liu B.C., Ju Y.L., Kang D.Z., Sun X.Y., Fang Y.L. (2020). Transcriptomics integrated with metabolomics reveals the effect of regulated deficit irrigation on anthocyanin biosynthesis in Cabernet Sauvignon grape berries. Food Chem..

[42] Paixao J., Dinis T.C.P., Almeida L.M. (2012). Protective role of malvidin-3-glucoside on peroxynitrite-induced damage in endothelial cells by counteracting reactive species formation and apoptoticmitochondrial pathway. Oxid. Med. Cell. Longev..

[43] Xu M., Chen X., Gu Y., Peng T., Yang D., Chang R.C.-C., So K.-F., Liu K., Shen J. (2013). Baicalin can scavenge peroxynitrite and ameliorate endogenous peroxynitrite-mediated neurotoxicity in cerebral ischemia-reperfusion injury. J. Ethnopharmacol..

[44] Shin M., Park E., Lee H. (2019). Plant-inspired pyrogallol-containing functional materials. Adv. Funct. Mater..

[45] Bita M., Preda M. (2004). The effect of polyphenols from *Vitis vinifera* on the oxidation of coffee lipids. Riv. Ital. Sostanze Gr..

[46] Mendes V., Vilaca R., de Freitas V., Ferreira P.M., Mateus N., Costa V. (2015). Effect of myricetin, pyrogallol, and phloroglucinol on yeast resistance to oxidative stress. Oxid. Med. Cell. Longev..

[47] Thi H.N., Waterhouse A.L. (2021). Redox cycling of iron: Effects of chemical composition on reaction rates with phenols and oxygen in model wine. Am. J. Enol. Viticult..

