# Supplementary material for: Metabolomics Integrated with HPLC–MS Reveals the Crucial Antioxidant Compounds of Muscadine Wine
Source: Antioxidants (Basel). 2022 Dec 27;12(1):55. doi: 10.3390/antiox12010055 (PMC9854500; doi:10.3390/antiox12010055)
Supplement: Supplementary file 1 [file antioxidants-12-00055-s001.zip › antioxidants-2057261-SI.pdf]

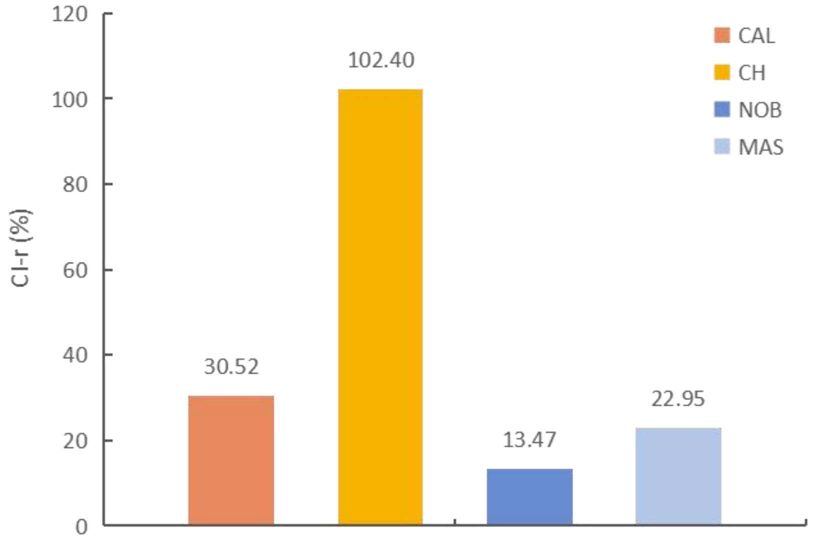

Figure S1. Colour intensity change rate (CI–r) during oxidation.

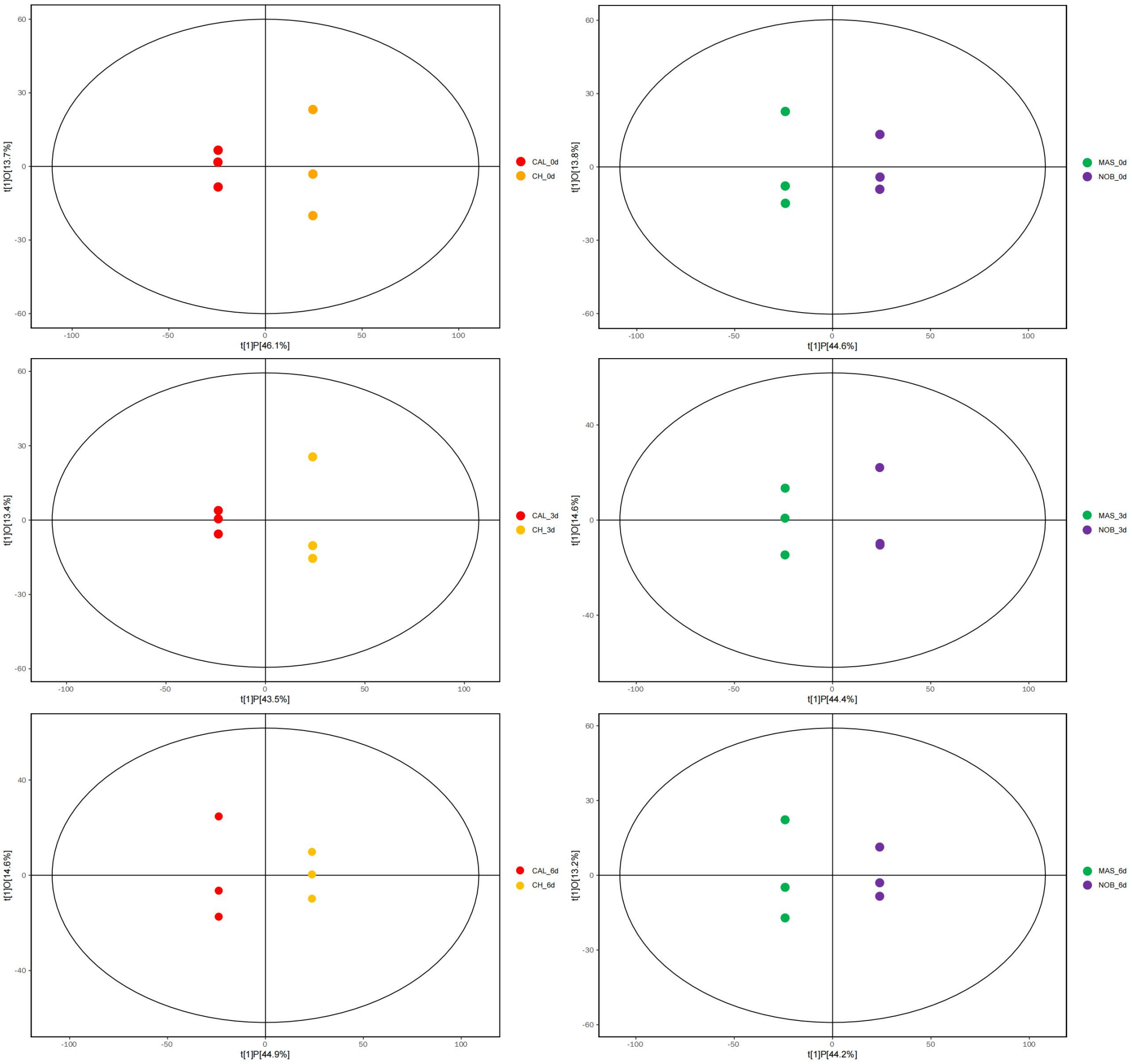

Figure S2. OPLS–DA score plots in different sample groups.

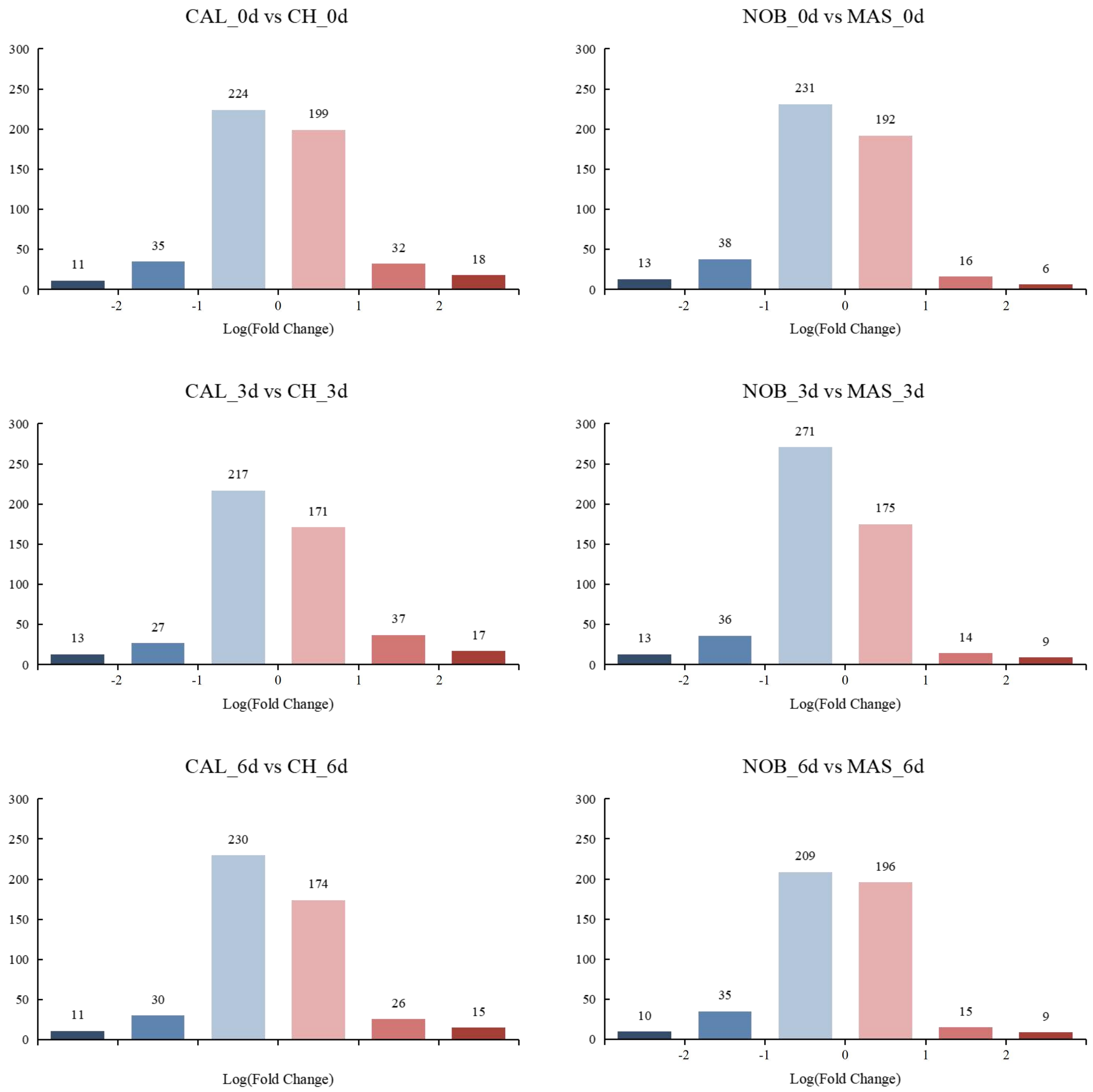

Figure S3. Fold change distribution of SCMs in different comparison group.

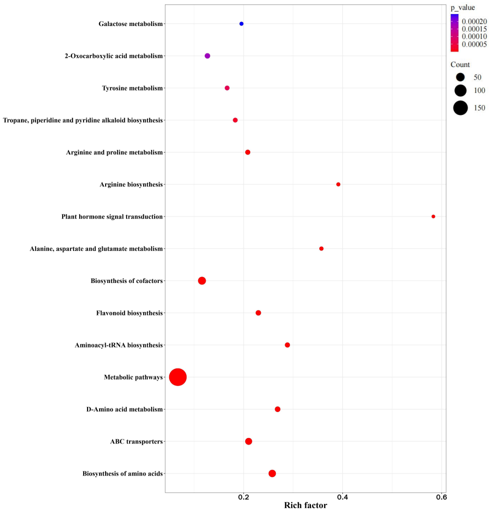

CAL_0d vs CH_0d

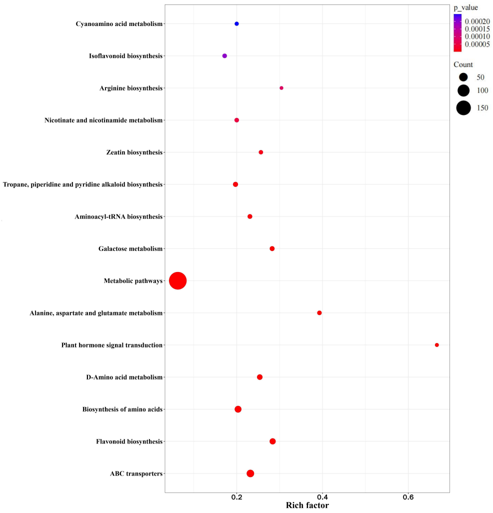

NOB_0d vs MAS_0d

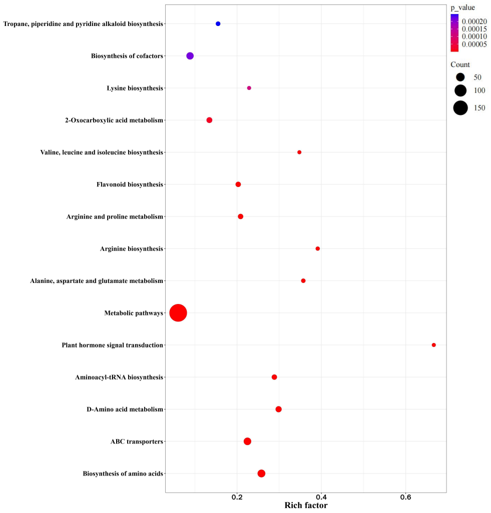

CAL_3d vs CH_3d

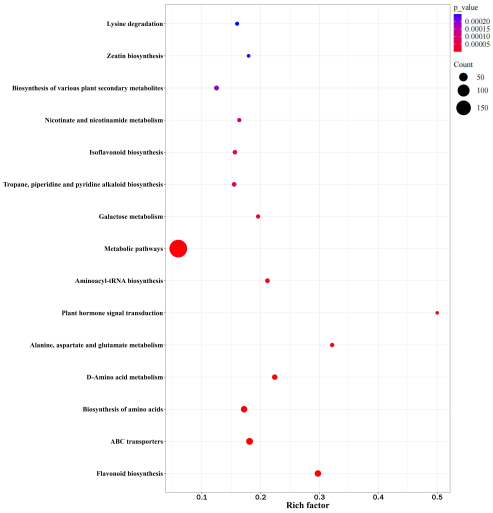

NOB_3d vs MAS_3d

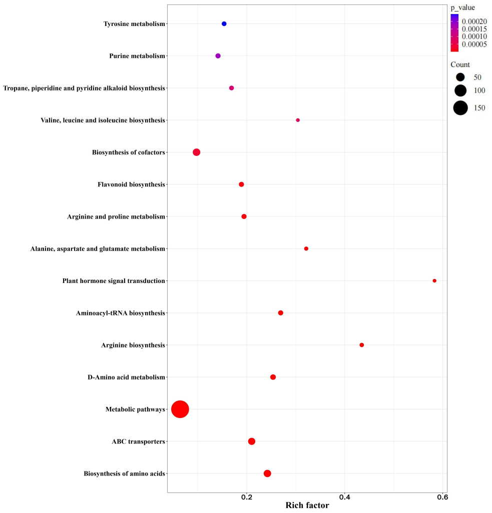

CAL_6d vs CH_6d

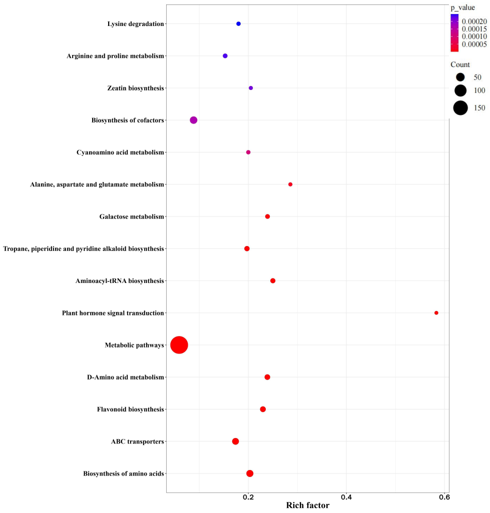

NOB_6d vs MAS_6d

Figure S4. KEGG enrichment for different comparison groups.

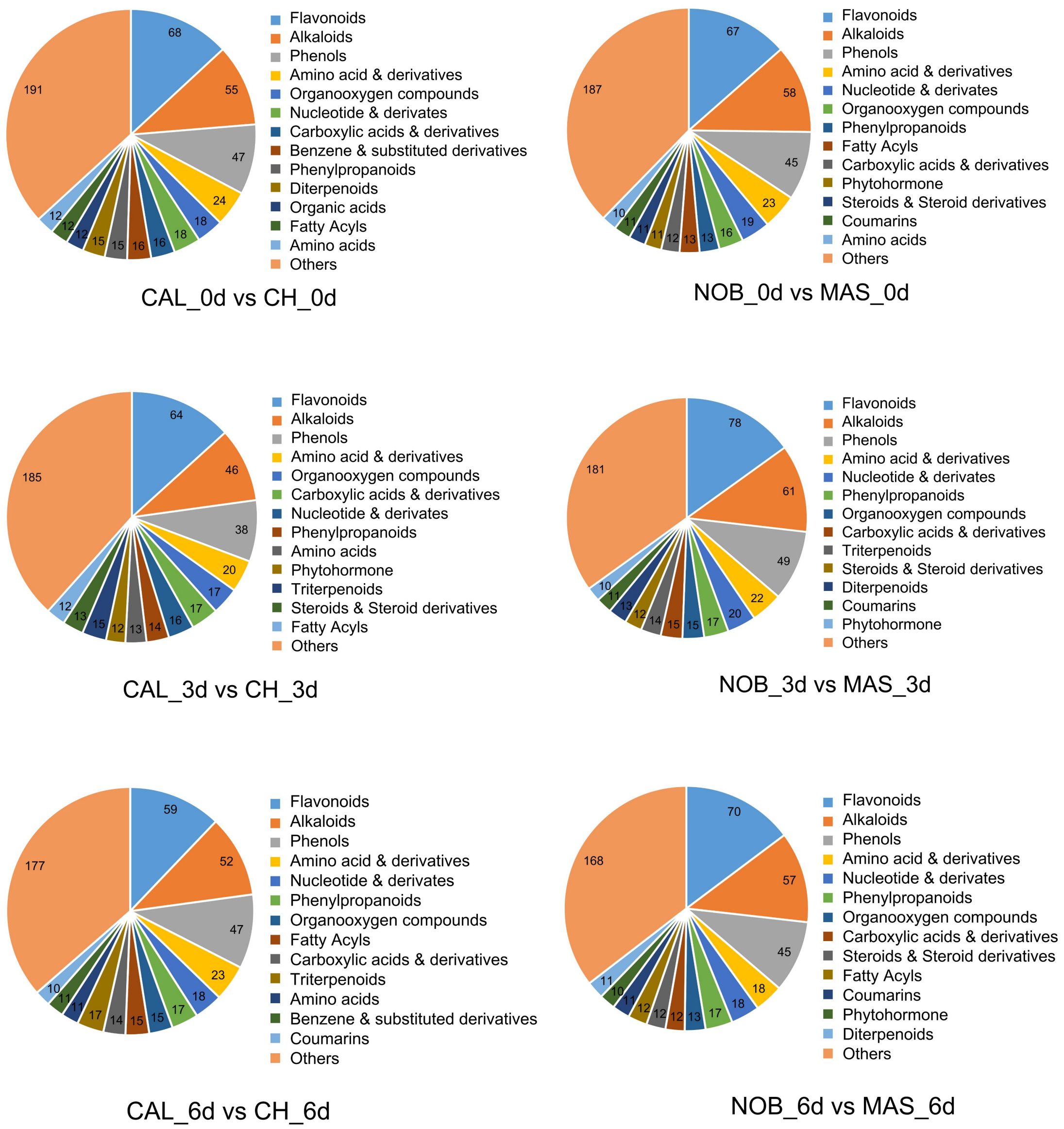

Figure S5. Classification of SCMs in different comparison groups.

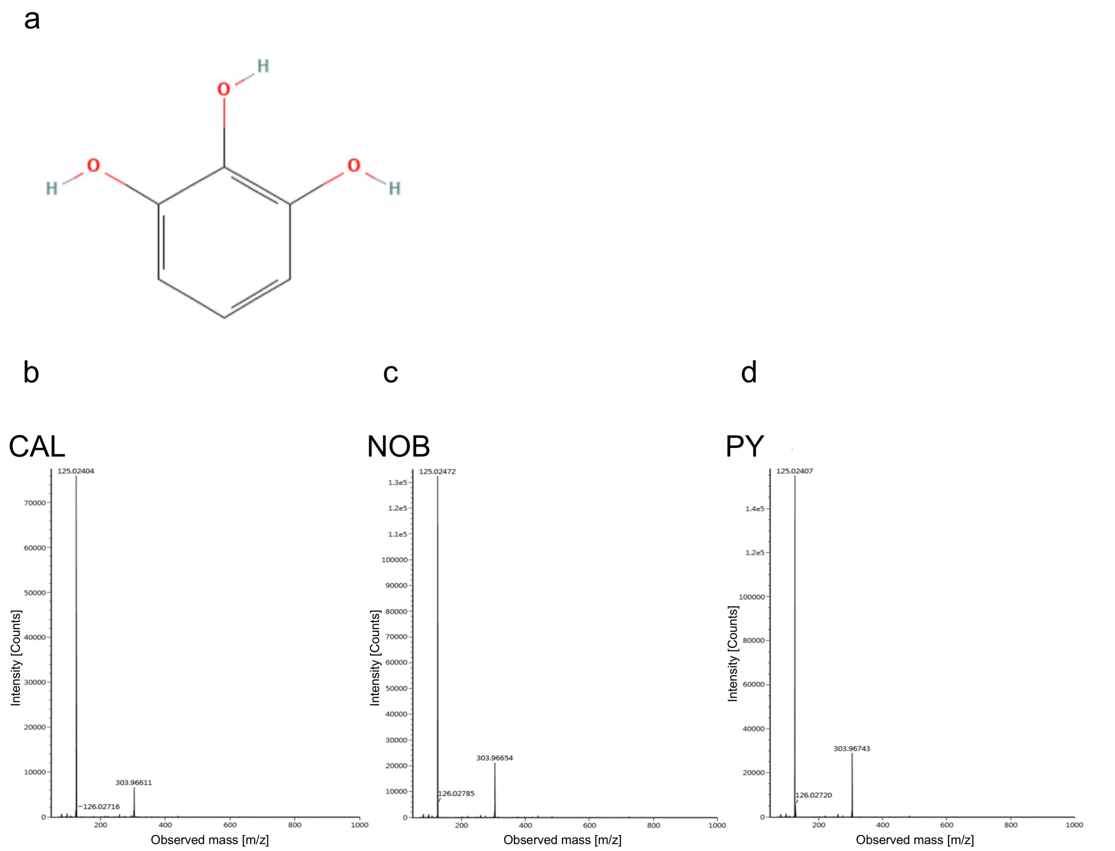

Figure S6. Mass spectrum information of wine and pyrogallol solution. (a) Chemical structue of pyrogallol; (b) Mass spectrum of the corresponding peak in CAL; (c) Mass spectrum of the corresponding peak in NOB; (d) Mass spectrum of the corresponding peak in pyrogallol.

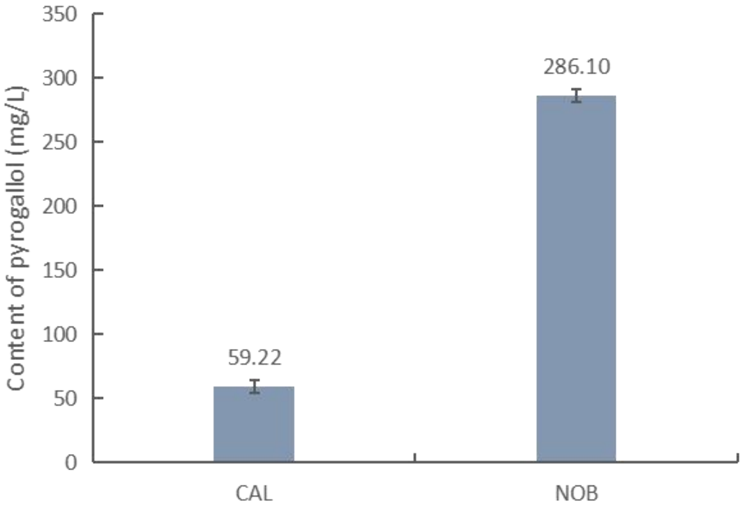

Figure S7. Contents of pyrogallol in CAL and NOB wine sample.
